# Longitudinal study of diet digestibility, microbiome, and gut fermentation metabolites in growing dogs

**DOI:** 10.1093/jvimsj/aalag001

**Published:** 2026-02-12

**Authors:** Eduarda L Fernandes, Renata B M S Souza, Lorenna N A Santos, Laiane S Lima, Heloísa L Silva, Simone G Oliveira, Ananda P Félix

**Affiliations:** Department of Animal Science, Federal University of Paraná, Rua dos Funcionários, 1540, Curitiba, Paraná 80035-050, Brazil; Department of Animal Science, Federal University of Paraná, Rua dos Funcionários, 1540, Curitiba, Paraná 80035-050, Brazil; Department of Animal Science, Federal University of Paraná, Rua dos Funcionários, 1540, Curitiba, Paraná 80035-050, Brazil; Department of Animal Science, Federal University of Paraná, Rua dos Funcionários, 1540, Curitiba, Paraná 80035-050, Brazil; Department of Animal Science, Federal University of Paraná, Rua dos Funcionários, 1540, Curitiba, Paraná 80035-050, Brazil; Department of Animal Science, Federal University of Paraná, Rua dos Funcionários, 1540, Curitiba, Paraná 80035-050, Brazil; Department of Animal Science, Federal University of Paraná, Rua dos Funcionários, 1540, Curitiba, Paraná 80035-050, Brazil

**Keywords:** age, consumption, intestinal functionality, puppies

## Abstract

**Background:**

Puppies have particular characteristics due to gastrointestinal immaturity, influencing physiological processes.

**Hypothesis/Objectives:**

Evaluate the effects of age and consumption on apparent diet digestibility coefficients (ADC), fecal characteristics, fermentation metabolites, and fecal microbiome in growing dogs.

**Animals:**

Eight dogs were evaluated at 2, 5, 8, 11, and 14 months old in experiment 1 and 12 dogs at 14 months old in experiment 2.

**Methods:**

This was a prospective experimental study. Dietary ADC, fecal characteristics, fermentation metabolites, and fecal microbiome were evaluated in 2 experiments: the first analyzed the effect of age, and the second, the effect of consumption in 2 groups: adult intake (AI) and puppy intake (PI).

**Results:**

Older dogs had lower ADC of dry matter (DM), ether extract, and metabolizable energy, and higher ADC of crude protein (CP) (*P* < .05). Younger dogs had lower fecal DM and fecal score and higher fecal production (*P* < .05). Older dogs had higher fecal concentrations of short-chain fatty acids and indoles and lower concentrations of branched-chain fatty acids (BCFA) (*P* < .05). Dogs aged between 2 and 5 months had a higher fecal abundance of *Streptococcus* and *Escherichia coli* and lower abundance of *Turicibacter* and *Peptacetobacter* (*P* < .05). The ADC of DM, organic matter, and CP were lower in PI dogs than in AI dogs (*P* < .05). The AI dogs had higher fecal DM and fecal score and lower fecal production (*P* < .05). Puppy intake dogs had higher fecal concentrations of ammonia and BCFA and a higher abundance of *Streptococcus* and a lower abundance of *Blautia* (*P* < .05).

**Conclusions and clinical importance:**

Age and feed intake influence the ADC of nutrients and energy, the fecal microbiome, and fermentation metabolites, with the microbiota stabilizing after 8 months of age in dogs.

## Introduction

Knowledge about nutrient use during dogs’ growth phase is essential to ensure health, proper development, and longevity. Most digestibility trials and microbiome or metabolome analyses in dogs evaluate the effects of dietary additives or ingredients without considering the potential influence of age or feed intake.^1–3^ Published studies comparing nutrient digestibility between adult dogs and puppies have drawn conflicting conclusions regarding nutrient utilization and production of intestinal fermentation metabolites.^2^^,^^4^^,^^5^

Puppies have some important peculiarities regarding their gastrointestinal physiology. Their gastrointestinal system is still developing after weaning, and the intestinal microbiota is immature,^6^^,^^7^ characterized by a higher abundance of *Streptococcus* and *Escherichia coli* and a lower abundance of *Faecalibacterium*, *Turicibacter*, and *Peptacetobacter hiranonis*. This microbial profile might influence the production of key metabolites and vitamins derived from bacterial fermentation.^6^

In addition, puppies have a higher energy demand to meet the growth needs as recommended by the National Research Council^8^ and the European Pet Food Industry Federation.^9^ This results in a proportionally higher food intake per kilogram of metabolic weight than adult dogs, especially when fed a moderate calorie-density diet. If an adult dog was fed a daily caloric load similar to that recommended for a puppy, the resulting increase in energy and nutrient intake, as well as in the passage rate, could reduce diet digestibility and the production of intestinal fermentation metabolites.^2^^,^^10^^,^^11^ However, it remains unclear whether feeding puppies an amount of calories appropriate for their life stage leads to similar changes in digestibility and intestinal fermentation.

Studying dogs’ growth phase allows us to identify the critical transition point from which diet digestibility, as well as variables such as the composition and diversity of the fecal microbiota and its fermentation metabolites, stabilize, and become comparable to those observed in adult dogs. The study aimed to evaluate the effects of age and feed intake on the apparent digestibility coefficients (ADC) of the diet, fecal characteristics, fermentation metabolites, and fecal microbiome at different stages of the dogs’ growth.

## Materials and methods

The use of animals in this study was approved by the Animal Use Ethics Committee of the Agricultural Sciences Sector of the Federal University of Paraná, Curitiba, PR, Brazil, under protocol no. 035/2022. The study occurred at the Laboratory of Studies in Canine Nutrition—LENUCAN in Curitiba, Paraná, Brazil (25° 25′ 40″ S, 49° 16′ 23″ W).

### Experiment 1

#### Animals and facilities

Eight Beagle dogs (4 males and 4 females) were evaluated at 2 (56 ± 17.90 days), 5 (147 ± 17.90 days), 8 (238 ± 17.90 days), and 11 months of age (329 ± 17.90 days) and 6 of them (3 males and 3 females) were also evaluated at 14 months of age (424 ± 17.90 days). Mean body weight (BW) were 5.27 ± 1.48 kg at 2, 7.83 ± 1.47 kg at 5, 9.61 ± 0.61 kg at 8, 10.10 ± 0.61 kg at 11, and 10.65 ± 0.90 kg at 14 months. The 2 dogs not evaluated at 14 months of age were reallocated to make up the group for the second experiment. All animals underwent prior clinical evaluations and were considered healthy. The clinical evaluation consisted of physical examination, blood analysis (complete blood count and biochemical profile), and fecal examination (flotation and SNAP tests to analyze giardia, coccidia, parasite eggs, and parvovirus).

The dogs were individually housed in brick kennels (5 m long × 2 m wide), with a bed and free access to fresh water. The facilities had bars on the side walls that allowed limited visual interaction with neighboring dogs, and there was additional environmental enrichment within the kennel during this period. The ambient temperature ranged from 16°C to 28°C, with a 12 h light–dark cycle (light from 6:00 am to 6:00 pm).

During the adaptation and collection periods, the dogs were kept exclusively inside the kennels. Outside of these times, they had daily access to a 1137 m^2^ outdoor area for 4 h for voluntary exercise and socialization. All dogs followed the same standardized daily routine and were treated by the same team throughout the study. The study was conducted between September 2022 and September 2023.

#### Food management

Dogs received the same commercial extruded dry food for growing dogs during the study. The dogs were weighed weekly and fed twice a day (at 08:00 am and 4:00 pm) according to their metabolizable energy requirements (MER) for growth or maintenance (depending on age) recommended by FEDIAF (2024)^9^ and adjusted according to the proposed growth curve for healthy puppies.^12^ The equation used was: MER (kcal/day) = [254.1 − 135.0 × (current BW, kg/expected adult BW, kg)] × current BW, kg^0.75^.^9^

The diet (Table 1) contained the following main ingredients: meat and bone meal, rice bran, poultry viscera meal, corn, wheat bran, corn gluten, and chicken oil.

**Table 1:** Analyzed chemical composition of the experimental diet based on dry matter (%).

Item	Diet, %
**Dry matter**	94.34
**Crude protein**	24.16
**Acid hydrolyzed ether extract**	11.74
**Ash**	10.52
**Total dietary fiber**	6.43
**Calcium**	2.44
**Phosphorus**	1.68
**Gross energy (Kcal/kg)**	4601.85

#### Digestibility and fecal characteristics

The digestibility test followed the total feces collection method recommended by the Association of American Feed Control Officials (AAFCO) (2016),^13^ with at least 15 days of adaptation, followed by 5 days of total feces collection per evaluation period.

Feces were collected at least twice daily for 5 days at 2, 5, 8, 11, and 14 months. They were stored in previously identified individual plastic containers, capped, and kept frozen at −14°C for posterior analysis.

At the end of the collection period, the feces were thawed at room temperature and homogenized separately, forming a composite sample from each animal. They were dried in a forced ventilation oven (320-SE, Fanem, São Paulo, Brazil) at 55°C for 72 h or until they reached a constant weight. After drying, the feces and experimental diet were ground using a 1 mm sieve in a grinder (Arthur H. Thomas Co., Philadelphia, PA, USA) and analyzed for dry matter (DM) at 105°C for 12 h, crude protein (CP, nitrogen × 6.25, method 954.01), mineral matter (method 942.05), and ether extract in acid hydrolysis (AEE, method 942.05). All analyses followed the Association of Official Analytical Chemists’ recommendations.^14^ Total dietary fiber (TDF) analysis in the diet occurred according to Prosky, Asp, Schweizer, Devries & Furda (1988).^15^ Gross energy (GE) was determined using a bomb calorimeter (IKA C2000 Basic, IKA-Werke, Staufen, Germany).

Fecal characteristics evaluation occurred during the collection period and included DM content (fecal dry matter [FDM]), production, score, and pH analysis. The fecal score was always determined by the same researcher, assigning points from 1 to 5, in which: 1 = soft and unformed feces to 5 = well-formed, hard, and dry feces, according to Carciofi et al. (2009).^16^ Fecal pH was measured with a digital pH meter (331, Politeste Instrumentos de Teste Ltda., São Paulo, SP, Brazil) using 3.0 g of fresh feces diluted in 30 mL of distilled water.

#### Fermentation metabolites and fecal microbiota

On the days the dogs reached 2, 5, 8, 11, and 14 months of age, fresh fecal samples were collected individually, after a maximum of 15 min of defecation, for ammonia, phenols, indoles, biogenic amines, short-chain fatty acids (SCFA), branched-chain fatty acids (BCFA), and microbiota analyses. Only fecal microbiota was assessed at 3 months of age.

Fecal ammonia concentration was determined according to Brito et al. (2010).^17^ For the determination of SCFA and BCFA, 10 g of fecal sample were weighed and mixed with 30 mL of 16% formic acid. The solutions were centrifuged at 2500 rpm (2 K15, Sigma, Osterode am Hans, NI, Germany) for 15 min. At the end of centrifugation, the supernatant was separated and centrifuged 3 times. Fecal SCFA and BCFA were analyzed by gas chromatography (Shimadzu, model GC-2014, Kyoto, Honshu, Japan), using a 30 m × 0.32 mm glass column (Agilent Technologies, HP INNO wax—19.091 N, Santa Clara, CA, United States of America). Nitrogen was used as the carrier gas with a 3.18 mL/min flow rate.

Phenols and indoles were analyzed with a GCMS2010 Plus gas chromatograph (Shimadzu) coupled to a TQ8040 mass spectrometer with an AC 5000 auto amplifier and a splitless injector. Chromatographic separations were obtained on the SH-Rtx-5MS column (30 m × 0.25 mm × 0.25 μm—Shimadzu) with a flow rate of 1.0 mL min^−1^ and helium as carrier gas at 5.0 speed. The transfer line and ionization source temperatures continued at 40°C and 220°C, respectively, and the 1 L injection volume remained in split mode (ratio 1:10). The GC oven temperature continued at 220°C (5 min), with an increase of 40°C min^−1^ to 280°C (5 min). The analysis lasted 31 min, and the mass spectrometer operated in full scan (*m*/*z* = 40-400) and selective ion monitoring modes, electron ionization at 70 eV. Data analysis employed GCMSsolution software.

To evaluate the fecal microbiota, approximately 2 g were removed from the interior of freshly collected feces, placed in a sterile Eppendorf microtube, and stored in a freezer at −80°C until analysis. DNA was extracted from the samples using the commercial ZR Fecal DNA MiniPrep kit (Zymo Research, Irvine, CA), following the manufacturer’s recommended protocol. The extracted DNA was quantified by spectrophotometry at 260 nm using the NanoDrop 2000 spectrophotometer (ThermoScientific). A 460-base segment of the V3V4 hypervariable region of the 16S rRNA ribosomal gene was amplified using the universal primers 515F and 806R and the following PCR conditions: 95°C for 3 min; 25 cycles of 95°C for 30 s, 55°C for 30 s, and 72°C for 30 s; followed by 72°C for 5 min. The metagenomic library was constructed from the amplified segments using Illumina’s commercial “Nextera DNA Library Preparation Kit.” They were pooled and sequenced on the Illumina “MiSeq” sequencer.^18^ The obtained reads were analyzed on the QIIME2 (Quantitative Insights Into Microbial Ecology) platform^19^^,^^20^; low-quality sequences and chimeras were removed, and taxonomic classification was carried out. In addition, potential contaminant amplicon sequencing variants (ASVs) were identified based on their prevalence in negative controls and were filtered from the entire dataset using the decontam R package. The sequences were classified into bacterial genera through the recognition of ASVs. Sequence comparison used the 2021 GTDB 202 update of the Genome Taxonomy Database ribosomal sequence database.^21^

A total of 12 293 reads per sample were used to generate the classification of bacterial communities by ASV identification to normalize the data.

#### Calculations and statistical analysis

The ME was estimated according to AAFCO (2016)^13^: ME (kcal/g) = {(GE of food consumed − GE excreted in feces) − [(CP consumed − CP excreted in feces) × 1.25]}/DM intake. Based on the obtained laboratory results, the apparent digestibility coefficients (ADC, %) of nutrients and energy were determined according to the equation: ADC (%) = [(ingested nutrient − excreted nutrient)/ingested nutrient] × 100.

Using the G*Power (version 3.1.9.7) statistical analysis software, a priori sample size was calculated based on effect size = 0.83, power = 0.80, and α = 0.05. The effect size was calculated using means and standard deviation (calculated from standard error of means) values reported in previous studies for the ADC of DM, fecal ammonia concentrations,^1^^,^^4^^,^^5^ and fecal microbiota α-diversity measures^22^ comparing adult and puppy dogs. The computed sample size was a total of 5 animals per time point. However, considering the recommendations of at least 6 dogs/treatment in nutritional trials by AAFCO (2016)^13^ and other studies with dogs^1^^,^^2^^,^^17^ we adopted 8 animals/time point. Initially, the digestibility data, fecal characteristics, and fermentation metabolites were analyzed using the Shapiro–Wilk normality test. Since the initial premises were met, a linear mixed model was applied to analyze the data, considering the age as a fixed effect and individual dogs as random effects. Linear and quadratic effects were tested using the lme4 package of R (version 4.3.1).

Fecal microbiome was analyzed by linear discriminant analysis of effect size (LEfSe), with a probability value adjusted for false discovery rate < .05. Alpha-diversity index data (Shannon, Chao1, and ASVs) were analyzed using the Kruskal–Wallis test (*P* < .05) and medians were compared by Dunn’s test. Beta-diversity was measured by principal coordinate analysis (PCoA), using the Bray–Curtis dissimilarity method. The differences between the general microbiota profiles among ages were analyzed by the PERMANOVA (permutational multivariate analysis of variance) test, considering *P* < .05. All the microbiota statistical analysis was conducted in MicrobiomeAnalyst (version 2.0) and the details about the methods can be found in Lu et al.^23^

### Experiment 2

#### Animals and facilities

The same 8 Beagle dogs (4 males and 4 females) from experiment 1 at 14 months (424 ± 17.90 days) were used. Four other Beagle dogs (2 males and 2 females) at 14 months (424 ± 17.90 days), with an average BW of 10.59 ± 0.98 kg, were added to experiment 2, totaling 12 dogs. They had a body condition score of 4-5, on a scale of 1-9.^24^ All animals underwent prior clinical evaluations and were considered healthy. The clinical evaluation consisted of physical examination, blood analysis, and fecal examination, as described in experiment 1. Dogs were kept in the same facilities described in experiment 1.

#### Experimental groups and evaluations

Two experimental groups were evaluated: adult dogs with adult intake (AI) and adult dogs with puppy intake (PI). Six dogs were randomly assigned in each experimental group (3 males and 3 females/group) using the “RAND” function of excel (Microsoft Excel, version 2509, 2019). The animals were fed the same commercial dry diet formulated for growth used in experiment 1 during 15 days of adaptation before fecal collection, in accordance with AAFCO (2016)^13^ recommendations.

Adult intake dogs received an amount of food of approximately 130 kcal/kg^0.75^/day to meet their MER for maintenance, according to the NRC (2006)^8^ and the animals’ history. Puppy intake dog’s intake was approximately 210 kcal/kg^0.75^/day, the same amount in g of DM per kg^0.75^ as the 2-month-old dogs in experiment 1. Animals received water freely.

The groups’ dietary ADC and ME, fecal characteristics, fermentation metabolites, and fecal microbiota were evaluated, as described for experiment 1.

#### Calculations and statistical analysis

The same calculations described in experiment 1 were used. The only difference was the use of 6 animals per experimental group, due to the availability of only 12 dogs in total for experiment 2. Data were first analyzed using the Shapiro–Wilk normality test. Data with normal distribution were analyzed using Student’s *t*-test (*P* < .05), considering a completely randomized design. Nonparametric data were analyzed by Mann–Whitney test (*P* < .05). Fecal microbiota data underwent the same analyses described in experiment 1.

## Results

### Experiment 1

#### Digestibility and fecal characteristics

No adverse reactions to the diet, such as vomiting or diarrhea, were observed, and all dogs consumed the entire amount of food offered throughout the experimental period. There was a quadratic behavior in DM, organic matter (OM), CP, TDF, AEE, and ME intake per kg^0.75^/day, with lower values starting at 8 months of age (*P* < .001, Table 2). Age did not alter the ADC of OM (*P* = .103) and GE (*P* = .059, Table 2). The ADC of DM decreased linearly (*P* < .001) and of CP increased linearly (*P* = .001) from 2 to 14 months of age (Table 2). There was a quadratic behavior in the ADC of AEE, with higher values at 2 months of age (*P* < .001, Table 2). Age linearly increased FDM and fecal score (*P* < .001) and linearly decreased fecal production (*P* < .001, Table 2). However, it did not influence fecal pH (*P* = .916, Table 3).

**Table 2:** Experiment 1—means of intake (on dry matter basis), apparent digestibility coefficients (ADC), metabolizable energy (kcal/kg), and fecal characteristics of dogs under 14 months of age.

Item	Age, months					SEM	*P*-value
	2 *n* = 8	5 *n* = 8	8 *n* = 8	11 *n* = 8	14 *n* = 8		
**Intake, g/kg** ^**0.75**^**/day**							
**Dry matter**	59.8	59.8	42.1	37.9	37.9	1.65	<.001
**Organic matter**	53.9	53.8	37.9	34.3	34.3	1.49	<.001
**Crude protein**	14.4	14.4	10.2	9.1	9.1	0.41	<.001
**Ether extract**	7.0	7.0	4.9	4.5	4.5	0.20	<.001
**Total dietary fiber**	3.8	3.8	2.7	2.4	2.4	0.11	<.001
**Metabolizable energy**	210.1	207.5	144.6	129.9	127.7	6.18	<.001
**ADC, %**							
**Dry matter**	71.9	70.1	68.2	68.5	67.5	0.43	<.001
**Organic matter**	75.4	74.3	74.1	75.0	73.0	0.31	.103
**Crude protein**	76.0	75.7	77.3	79.3	77.1	0.31	.001
**Ether extract**	86.0	83.0	81.2	84.0	83.5	0.37	<.001
**Gross energy**	76.2	75.3	75.4	75.6	73.7	0.31	.059
**Metabolizable energy**	3512.7	3469.2	3434.3	3427.0	3370.2	15.00	.002
**Fecal characteristics**							
**Dry matter, %**	26.9	28.4	32.0	33.7	35.5	0.58	<.001
**Fecal production (g/day)**	269.2	313.4	231.7	201.2	202.0	7.64	<.001
**Fecal score**	3.0	3.1	3.6	3.8	3.9	0.09	<.001

Ether extract: in acid hydrolysis; SEM: pooled standard error of mean; *n*: number of dogs. *P*-values of the best-fitting model for age effects; linear responses to age: ADC of dry matter and crude protein, ME, fecal dry matter, fecal production, and fecal score; quadratic responses to age: intake of dry matter, organic matter, crude protein, ether extract, total dietary fiber, and metabolizable energy, and ADC of ether extract in acid hydrolysis.

**Table 3:** Experiment 1—means of pH and fermentation metabolites in feces of dogs under 14 months of age.

Item	Age, months					SEM	*P*-value
	2 *n* = 8	5 *n* = 8	8 *n* = 8	11 *n* = 8	14 *n* = 8		
**pH**	6.5	6.6	6.8	6.7	6.4	0.07	.916
**SCFA (μmol/g)**							
**Acetate**	63.4	76.4	176.1	142.0	135.0	8.21	<.001
**Propionate**	26.3	29.9	61.2	56.0	50.5	2.70	<.001
**Butyrate**	7.9	9.1	15.1	12.8	13.0	0.53	<.001
**Total SCFA**	97.6	112.0	242.9	210.8	198.5	10.90	<.001
**Valerate**	5.5	5.3	4.9	4.6	4.8	0.08	<.001
**BCFA and volatiles (μmol/g)**							
**Isovalerate**	5.9	5.7	5.3	5.6	5.1	0.09	.039
**Isobutyrate**	5.8	5.6	5.9	5.6	5.2	0.07	.019
**Total BCFA**	11.7	11.3	11.2	11.4	10.3	0.21	<.001
**4-Methylvalerate**	0.6	0.5	0.6	0.6	0.6	0.01	.067
**Hexanoic**	0.7	0.6	0.7	0.6	0.6	0.02	.006
**Heptanoic**	5.6	5.3	4.7	4.5	4.2	0.09	<.001
**Phenols and indoles (% peak area)**							
**Phenol**	1.0	3.2	1.0	0.9	2.0	0.28	.760
**Indole**	2.6	11.4	19.1	23.1	15.5	1.56	<.001
**P-cresol**	1.7	2.3	6.8	4.2	8.9	0.61	<.001
**Ammonia**	0.1	0.2	0.1	0.1	0.1	0.01	.097

Abbreviations: SCFA = short-chain fatty acids; BCFA = branched-chain fatty acids; SEM = pooled standard error of mean; *n* = number of dogs.

*P*-values of the best-fitting model for age effects; linear responses to age: total BCFA, heptanoic, and p-cresol; quadratic responses to age: acetate, propionate, butyrate, total SCFA, isovalerate, isobutyrate, hexanoic acid, and indole.

#### Fermentation metabolites and fecal microbiota

A quadratic response was observed in fecal concentrations of acetate, propionate, butyrate, and total SCFA (*P* < .001, Table 3), with higher values in dogs from 5 months of age. Fecal concentrations of isovalerate (*P* = .039), isobutyrate (*P* = .044), and hexanoic acid (*P* = .006) also demonstrated a quadratic response, with lower values in older dogs (Table 3). Valerate, heptanoic, and total BCFA decreased linearly in feces from 2 to 14 months of age (*P* < .001, Table 3). No effects of age on fecal ammonia (*P* = .097) and 4-methylvalerate (*P* = .067) concentrations were observed (Table 3).

Age differences for the percentages of phenol peaks in feces were not observed (*P* = .760, Table 3). However, there was a quadratic response in the percentages of indole peaks, with the highest values starting at 5 months of age (*P* < .001). The percentages of p-cresol peaks increased linearly in feces from 2 to 14 months of age (*P* < .001, Table 3).

**Figure 1:**
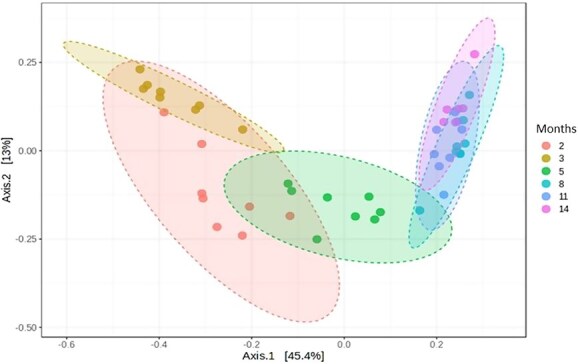
Experiment 1—β-diversity by Bray–Curtis dissimilarity method illustrating the differentiation of fecal bacterial communities of 8 dogs at 2-14 months of age. Each dot represents an animal and shaded ellipses indicate the grouping and dispersion of bacterial communities at each evaluated age. Bacterial communities differed at 2, 3, and 5 months of age (*P* < .001), but did not differ at 8, 11, and 14 months of age (*P* = .647). Bacterial communities differed between initial months (2, 3, and 5) when compared to late months of age (8, 11, and 14) by PERMANOVA test (*P* < .001). Abbreviation: PERMANOVA = permutational multivariate analysis of variance.

Beta-diversity analysis (Figure 1) demonstrated differences in fecal bacterial communities’ profile at 2, 3, and 5 months of age (*P* < .001) and between initial months (2, 3, and 5) when compared to late months of age (8, 11, and 14) (*P* < .001). The general fecal microbiota profile did not differ at 8, 11, and 14 months of age (*P* = .647).

Regarding α diversity, there was a reduction in the Chao1 index and ASVs at 3, 5, 8, 11, and 14 months compared to 2 months of age (*P* < .001). The Shannon index did not show any significant difference between the studied ages (*P* = .680, Table 4).

**Table 4:** Experiment 1—medians of α-diversity indexes (ASV’s, Chao1, and Shannon) of dogs under 14 months of age.

Item	Age, months						IQR	*P*-value
	2 *n* = 8	3 *n* = 8	5 *n* = 8	8 *n* = 8	11 *n* = 8	14 *n* = 8		
**Chao1**	318.08^a^	203.84^bc^	222.39^b^	200.51^bc^	167.77^c^	158.52^c^	23.36	<.001
**ASVs**	268.58^a^	190.25^bc^	200.82^b^	198.71^bc^	167.17^bc^	157.68^c^	15.94	<.001
**Shannon**	3.77^a^	3.79^a^	3.68^a^	3.77^a^	3.66^a^	3.59^a^	0.03	.680

Abbreviations: ASVs = amplicon sequencing variants; *n* = number of dogs; *P* = probability by Kruskal–Wallis test (*P* < .05).

Medians followed by different superscript letters a, b, c differ by Dunn’s test (*P* < .05).

**Figure 2:**
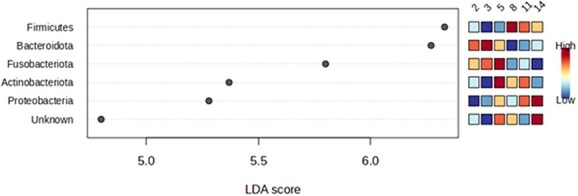
Experiment 1—LDA scores of the main bacterial phyla that differed in the feces of 8 dogs at different age points (2, 3, 5, 8, 11, and 14 months). Adjusted *P* < .001. Abbreviation: LDA = linear discriminant analysis.

The LEfSe analysis identified key bacterial phyla as significant biomarkers for different age groups (Figure 2). Bacteroidota and Fusobacteriota were the characteristic discriminant taxa of the younger ages (2 and 3 months), while at 5 months of age, Fusobacteriota and Actinobacteriota were more abundant (*P* < .001). At 8 months of age, Firmicutes emerged as the primary biomarker (*P* < .001). Later, at 11 and 14 months of age, Proteobacteria, Firmicutes, and Actinobacteriota were all identified as key discriminant phyla (*P* < .001).

**Figure 3:**
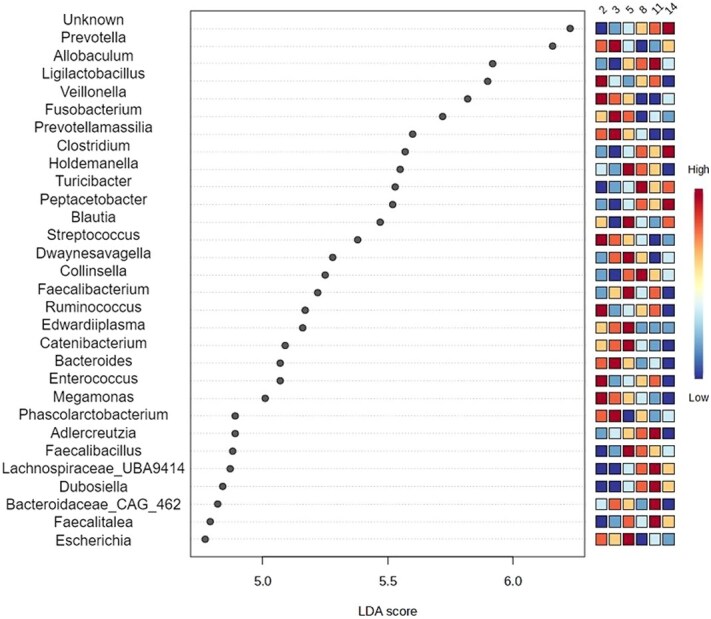
Experiment 1—LDA scores of the main bacterial genera that differed in the feces of 8 dogs at different age points (2, 3, 5, 8, 11, and 14 months). Adjusted *P* < .05. Abbreviation: LDA = linear discriminant analysis.

At the genus level, LEfSe analysis identified a range of taxa exhibiting differential enrichment across distinct age stages (Figure 3). Several genera were enriched at 2-5 months of age. These included *Ligilactobacillus* (*P* < .001), *Ruminococcus* (*P* < .001), and *Enterococcus* (*P* = .003) (2 months of age); *Prevotella* (*P* < .001) (2 and 3 months of age); and a larger group including *Veillonella* (*P* < .001), *Fusobacterium* (*P* < .001), *Prevotellamassilia* (*P* < .001), *Streptococcus* (*P* < .001), *Bacteroides* (*P* = .003), *Edwardiiplasma* (*P* < .001), *Catenibacterium* (*P* < .001), *Megamonas* (*P* < .001), and *Escherichia* (*P* < .001), in the entire 2- to 5-month period.

**Figure 4:**
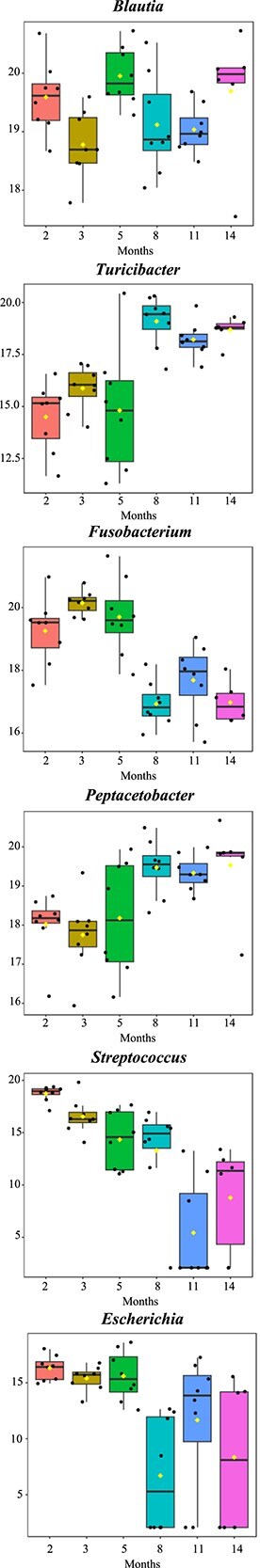
Experiment 1—abundance (log DNA) of the genera *Blautia*, *Turicibacter*, *fusobacterium*, *Peptacetobacter*, *Streptococcus*, and *Escherichia* in the feces of 8 dogs aged 2, 3, 5, 8, 11, and 14 months.

Conversely, other genera were enriched in later growth stages (5-14 months of age). Key discriminant taxa for this period included *Allobaculum* (*P* < .001), *Holdemanella* (*P* = .042), *Collinsella* (*P* < .001), *Adlercreutzia* (*P* < .001), and *Faecalibacillus* (*P* < .001) (8 and 11 months of age); *Clostridium* (*P* < .001), *Turicibacter* (*P* < .001), and *Peptacetobacter* (*P* = .002) (8-14 months of age); and *Faecalitalea* (*P* < .001) (5-14 months of age). Figure 4 (*P* < .05) presents the abundance of key taxa (*Fusobacterium*, *Turicibacter*, *Escherichia*, *Blautia*, and *Streptococcus*) in the feces of dogs aged 2-14 months.

### Experiment 2

#### Digestibility and fecal characteristics

No adverse reactions to the diet, such as vomiting or diarrhea, were observed, and all dogs consumed the entire amount of food offered throughout the experimental period. The DM, OM, CP, TDF, AEE, and ME intake per kg^0.75^/day was higher in the PI group when compared to AI dogs (*P* < .001, Table 5). The ADC of DM (*P* = .003), OM (*P* = .006), CP (*P* = .017), and GE (*P* = .017) were lower for PI dogs compared to AI (Table 5). In contrast, the ADC of AEE (*P* = .283) and the ME (*P* = .190) did not differ between groups (Table 5). Fecal dry matter and fecal score were higher (*P* = .012) and fecal production (*P* < .001) was lower in AI dogs when compared to PI dogs (Table 5).

**Table 5:** Experiment 2—means of intake (on dry matter basis), apparent digestibility coefficients (ADCs), metabolizable energy, and fecal characteristics of dogs in the adult intake (AI) and puppy intake (PI) groups.

Item	Groups		SEM	*P*-value
	AI *n* = 6	PI *n* = 6		
**Intake, g/kg** ^**0.75**^**/day**				
**Dry matter**	37.9	59.9	3.32	<.001
**Organic matter**	33.6	54.0	3.07	<.001
**Crude protein**	9.1	14.7	0.83	<.001
**Ether extract**	4.4	7.0	0.40	<.001
**Total dietary fiber**	2.4	3.9	0.22	<.001
**Metabolizable energy, kcal**	127.7	196.4	13.20	<.001
**ADC, %**				
**Dry matter**	67.5	65.7	0.34	.003
**Organic matter**	73	71.0	0.38	.006
**Crude protein**	77.1	74.2	0.60	.017
**Ether extract in acid hydrolysis**	83.5	82.5	0.43	.283
**Gross energy**	73.7	71.7	0.44	.017
**Metabolizable energy (kcal/kg)**	3370.2	3279.1	18.60	.190
**Fecal characteristics**				
**Dry matter, %**	35.5	33.1	0.51	.012
**Fecal production (g/dog/day)**	202	363.7	25.30	<.001
**Fecal score**	3.9	3.4	0.10	.012

Ether extract: in acid hydrolysis; SEM: pooled standard error of mean; *n*: number of dogs; *P*: probability by Student’s *t*-test (*P* < .05).

#### Fermentation metabolites and fecal microbiota

Fecal ammonia concentrations were higher in PI dogs compared to AI dogs (*P* = .021, Table 6). Higher fecal concentrations of butyrate (*P* = .012), isobutyrate (*P* = .002), valerate (*P* = .001), hexanoic (*P* = .009), heptanoic (*P* = .008), and total BCFA (*P* = .005) were observed in PI dogs compared to AI dogs (Table 6). There was no significant difference in fecal pH (*P* = .074), acetate (*P* = .936), propionate (*P* = .351), total SCFA (*P* = .936), isovalerate (*P* = .382), and 4-methylvalerate (*P* = .106, Table 6). No differences were found between groups in the peak percentages area of phenols (*P* = .396), indoles (*P* = .361), and p-cresols (*P* = .952, Table 6).

**Table 6:** Experiment 2—Means of pH and fermentation metabolites in the feces of dogs in the adult intake (AI) and puppy intake (PI) groups.

Item	Groups		SEM	*P*-value
	AI *n* = 6	PI *n* = 6		
**pH**	6.40	6.7	0.09	.074
**SCFA (μmol/g dry matter)**				
**Acetate**	135.0	136.0	5.77	.936
**Propionate**	50.5	46.6	1.98	.351
**Butyrate**	13.0	14.6	0.35	.012
**Total SCFA**	198.5	197.2	7.38	.936
**Valerate**	4.8	6.2	0.25	.001
**BCFA and volatiles (μmol/g dry matter)**				
**Isovalerate**	5.1	5.3	0.09	.382
**Isobutyrate**	5.2	5.7	0.08	.002
**Total BCFA**	10.3	11.0	0.24	.005
**4 methyl valerate**	0.6	0.6	0.01	.106
**Hexanoic**	0.6	0.8	0.04	.009
**Heptanoic**	4.2	4.6	0.07	.008
**Phenols and indoles (% peak area)**				
**Phenol**	1.3	2.0	0.37	.396
**Indole**	18.2	15.5	1.39	.361
**P-cresol**	9.1	8.9	1.26	.952
**Ammonia**	0.1	0.2	0.02	.021

Abbreviations: SCFA = short-chain fatty acids; BCFA = branched-chain fatty acids; SEM = pooled standard error of mean; *n* = number of dogs; *P* = probability by Student’s *t*-test.

**Figure 5:**
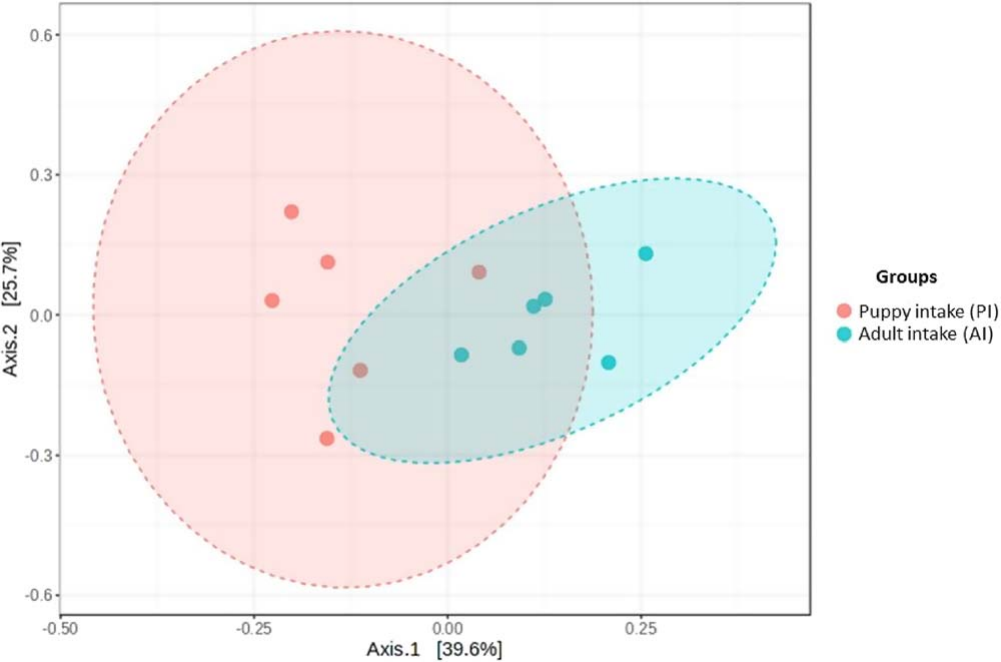
Experiment 2—β-diversity by Bray–Curtis dissimilarity method illustrating the differentiation of bacterial communities of 6 dogs in the AI group and 6 dogs in the PI group. Each dot represents an animal and shaded ellipses indicate the grouping and dispersion of bacterial communities at each group. *P* = .032 by PERMANOVA test. Abbreviations: AI = adult intake; PERMANOVA = permutational multivariate analysis of variance; PI = puppy intake.

Beta-diversity analysis demonstrated general differences in fecal bacterial communities profile between the PI and AI groups (*P* = .032, Figure 5). Regarding α diversity, the number of ASVs (*P* = .002), Chao1 (*P* = .003), and Shannon (*P* = .001) indexes were higher in the PI group compared to the AI group (Table 7).

**Table 7:** Experiment 2—medians of α-diversity indexes (ASVs, Chao1, and Shannon) of dogs in the adult intake (AI) and puppy intake (PI) groups.

Item	Groups		IQR	*P*-value
	AI *n* = 6	PI *n* = 6		
**Chao1**	152.1	185.0	6.75	.003
**ASVs**	151.3	184.0	6.55	.002
**Shannon**	3.3	3.7	0.06	.001

Abbreviations: ASVs = amplicon sequencing variants; *n* = number of dogs; *P* = probability by Mann–Whitney test.

**Figure 6:**
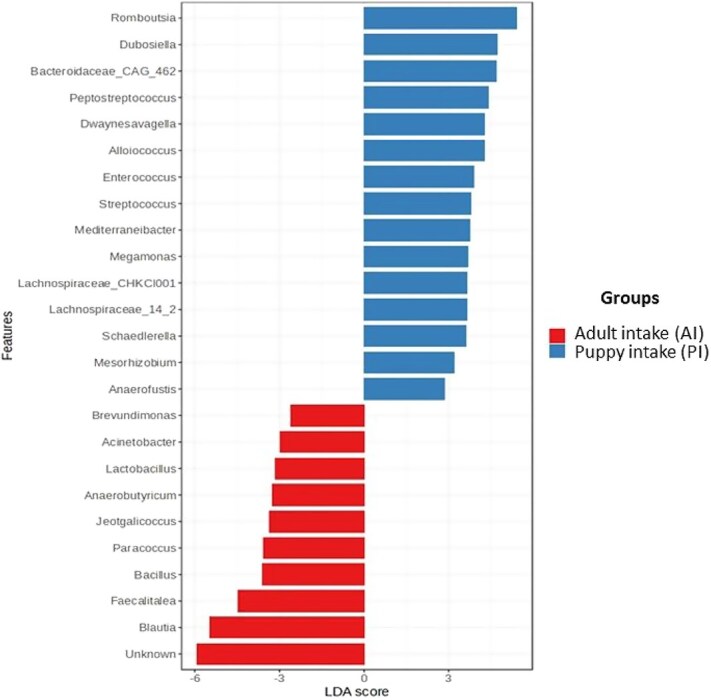
Experiment 2—LDA scores of the main bacterial genera that differed in the feces of 6 dogs in the AI group and 6 dogs in the PI group. Adjusted *P* < .05. Abbreviations: AI = adult intake; LDA = linear discriminant analysis; PI = puppy intake.

The LEfSe results showed that the main enriched genera in feces of PI group, compared to the AI group, were: *Romboutsia* (*P* < .001), *Dubosiella* (*P* < .001), *Bacteroidaceae_CAG_462* (*P* < .001), *Peptostreptococcus* (*P* < .001), *Dwaynesavagella* (*P* < .001), *Alloiococcus* (*P* = .002), *Enterococcus* (*P* < .001), *Streptococcus* (*P* < .001), *Mediterraneibacter* (*P* = .001), *Megamonas* (*P* < .001), *Lachnospiraceae_CHKCI001* (*P* < .001), and *Schaedlerella* (*P* = .039) (Figure 6). While the enriched genera in feces of the AI group compared to the PI group were: *Brevundimonas* (*P* = .041), *Acinetobacter* (*P* = .020), *Lactobacillus* (*P* = .001), *Anaerobutyricum* (*P* < .001), *Jeotgalicossus* (*P* < .001), *Paracoccus* (*P* < .001), *Bacillus* (*P* < .001), *Faecalitalea* (*P* < .001), and *Blautia* (*P* < .001) (Figure 6).

## Discussion

The dogs’ developmental period is critical for intestinal microbiota establishment, which is relevant for the gastrointestinal functionality. Few studies compare nutrient digestibility, fermentation metabolites, and intestinal microbiome between adult dogs and puppies, and the effect of food consumption on these variables. This study showed that age and food consumption can affect diet digestibility, fecal characteristics, fermentation metabolites, and the dogs’ intestinal microbiome.

As expected, younger dogs had higher consumption per kg^0.75^/day of nutrients and energy than adults. Growing dogs have more energy and nutritional demands than adult dogs. When a puppy reaches up to 50% of its adult BW, its caloric requirement is approximately 1.6 times higher than that observed in adult dogs.^9^

Due to higher consumption, experiment 2 aimed to verify whether the differences between adult dogs and puppies are due to the physiological stage or food intake. Some effects observed in puppies probably resulted from higher consumption and not just age, such as lower FDM, lower fecal score, and higher fecal production, which were similar between puppies in experiment 1 and adults in the PI group in experiment 2.

No studies were found that compared the same age range of puppies and adult dogs as in the present study. Most similar studies observed divergent results, reporting no age interference on the ADC of nutritional fractions or higher diet digestibility in adult dogs.^25^^,^^26^ Studies comparing the consumption effects on diet digestibility in adult dogs reported lower CP digestibility the higher its consumption.^27^^,^^28^^,^^29^^,^^30^^,^^31^ This can be explained because larger amounts of CP ingestion can exceed the absorptive capacity of dogs’ gastrointestinal tract, resulting in reduced digestibility.^10^ This was observed in younger dogs when compared to older dogs in experiment 1 and in dogs in the PI group when compared to those in the AI group in experiment 2. Lower CP digestibility was also observed in studies investigating dogs fed diets with high protein concentrations when compared to diets with lower concentrations.^27–31^

The higher ADC of AEE in younger than in older dogs may have contributed to the observed diet’s higher DM digestibility and higher ME. The higher apparent lipid digestibility observed in younger dogs may be attributed to the higher daily intake of AEE per kg^0.75^/day than that of adult dogs.^2^ Endogenous lipid losses contribute less to the apparent digestibility of AEE in adult dogs than in puppies, due to a lower proportion of lipid losses relative to intake in adults.^2^ This was also observed in other studies comparing the digestibility of AEE between adult dogs and puppies.^2^^,^^4^^,^^5^^,^^32^ As for experiment 2, when comparing 2 consumption quantities, no differences in ADC of AEE and the ME content of the diet were observed. This indicates the possibility that the greater lipid digestibility is specific to the digestion of puppies and independent of the evaluated food consumption levels.

Dietary digestibility can also modulate the intestinal microbiome of dogs during growth and adult stages. The establishment of the intestinal microbiome begins at birth and continues to evolve throughout the different stages of the dog’s life.^6^^,^^7^ As a result, bacterial communities continue to diversify after weaning, replacing important bacteria for milk digestion with others that take part in the digestion of complex diets.^7^

In this study, the fecal microbiota of dogs stabilized at 8 months of age, with changes observed throughout growth. The same was observed in other studies of growing dogs.^6^^,^^7^ In the first months of life, puppies have an immature intestinal microbiota, characterized by a higher abundance of *Streptococcus* and *E coli*, as observed in dogs up to 5 months of age.^6^ The decrease in the relative abundance of *E coli* throughout growth may indicate the transition from an aerobic environment to a sufficiently anaerobic environment.^22^ The shift to an anaerobic intestinal environment contributes to the growth of bacteria associated with eubiosis, such as *Turicibacter*, *Peptacetobacter* (*Clostridium hiranonis*), and *Blautia*,^33^ which increased in dogs starting at 8 months of age.

These differences in the microbiota profile interfere with the intestinal environment through different mechanisms; one is the alteration of fermentation metabolites produced. The greater fecal abundance of proteolytic bacteria, such as *Streptococcus* and *E coli* in the younger puppies, may be related to the higher concentrations of isobutyrate, isovalerate, hexanoic, and heptanoic acids, and total BCFA found in the fecal samples of this group. In high concentrations, these compounds can have inflammatory effects on the intestinal mucosa.^34–37^

In addition to the higher fecal concentrations of nitrogen metabolites, such as BCFA, puppies also presented lower fecal concentrations of SCFA. This result may be related to the lower abundance of SCFA-producing bacteria, such as *Turicibacter* and *Blautia*, observed in puppies.

Higher fecal SCFA concentrations observed in adult dogs may be associated with intestinal health.^36^ Short-chain fatty acids contribute to eubiosis of the intestinal microbiota, attenuate inflammation and oxidative stress, and participate in regular cell growth and differentiation.^38^^,^^39^ Acetate, the most abundant SCFA, is necessary for bacteria growth associated with intestinal eubiosis. Butyrate is considered one of the most essential fermentative metabolites for intestinal health and is the prime source of energy for colonocytes, helping to control inflammatory processes in the intestine.^40^^,^^41^

Increased fecal concentrations of indoles were observed in older dogs. Indoles play an important role in intestinal functionality, such as strengthening the mucosa and downregulating the expression of pro-inflammatory cytokines, and their effects (positive or negative) depend on their concentrations.^42^ Increased fecal concentrations of indoles in adult dogs could potentially be associated with positive effects, as these findings were observed alongside an increase in eubiosis-related bacteria and their metabolites in the present study.^42^ However, multiple factors may contribute to these changes, and the role of indoles should be interpreted within this broader context.

Although *Fusobacterium* is frequently associated with eubiosis in dogs,^33^ it is also recognized as a proteolytic genus.^43^ The decrease of *Fusobacterium* in older dogs may be associated with lower food consumption, resulting in increased CP digestibility, as both experiments have shown. This results in less available undigested substrate in the colon,^11^ reducing proteolytic genera abundance, including *Fusobacterium*.

Other genera were also reduced in the feces of adult dogs with lower CP intake (AI), when compared with the higher intake group (PI), such as *Peptostreptococcus*, *Enterococcus*, and *Streptococcus*. In addition, dogs in the AI group had lower fecal concentrations of nitrogen fermentation metabolites, such as ammonia, isobutyrate, hexanoic, and heptanoic acids, and total BCFA. There were also lower fecal butyrate concentrations in this group. Although butyrate is a metabolite associated with intestinal eubiosis and saccharolytic fermentation,^44^ some specific proteolytic bacteria, such as *Fusobacterium*, are also capable of producing this compound through the glutamate and lysine degradation pathways.^43^ Thus, this reduction in *Fusobacterium* abundance may therefore explain the lower fecal butyrate concentrations observed in the AI group.

Adult dogs with lower consumption (AI group) also had more *Blautia* in their feces. This genus is a well-known producer of SCFAs.^33^^,^^45^^,^^46^ No SCFA increase was observed in the AI group, likely due to the rapid absorption of these metabolites in the intestinal lumen before they reach the distal colon, which may reduce their concentrations in feces.^47^^,^^48^

Based on the results observed, highly digestible protein sources associated with a higher caloric diet density are recommended for growing dogs. This approach may improve the eubiosis of microbiota and reduce the production of metabolites with toxic potential for the intestinal mucosa.

This study has some limitations, such as the relatively small number of animals evaluated. While the use of a single diet enabled strict control over nutritional variables, it also restricts the extrapolation of results to other diets with different nutritional profiles. In addition, although diet, animal handling, and human contact were standardized throughout the study, seasonal and environmental variations during the experimental period may have influenced some results. Another limitation is that the microbiota characterization was based on 16S rRNA gene sequencing, which, although widely adopted, primarily offers taxonomic insights and limits functional interpretation of the fecal microbiota of dogs. Moreover, the inclusion of additional markers of gut functionality—such as calprotectin, bile acids, and intestinal permeability—could have enhanced the understanding of the observed outcomes. It is also important to note that this was a long-term study conducted under controlled kennel conditions, which may not fully reflect the environmental variability experienced by the general dog population.

## Conclusions

Younger dogs showed higher feed intake/kg BW^0.75^, ADC values of AEE and ME and lower ADC of CP compared to older dogs. Among adults, dogs in the PI group presented lower ADC of CP than those in the AI group. At approximately 8 months, dogs’ microbiota resembles that of healthy adults, remaining stable in adulthood, as they have a greater fecal abundance of genera related to intestinal eubiosis and higher concentrations of SCFA. In contrast, younger dogs have a reduced fecal abundance of genera related to intestinal eubiosis and higher concentrations of BCFA. These results offers insights into future nutritional strategies to improve intestinal functionality during growth.

## Off-label antimicrobial declaration

The authors declare no off-label use of antimicrobials.

## Institutional animal care and use committee or other approval declaration

Approved by the Animal Use Ethics Committee of the Agricultural Sciences Sector of the Federal University of Paraná, Curitiba, PR, Brazil, under protocol no. 035/2022.

## Abbreviations

AAFCO: Association of American Feed Control Officials
ADC: apparent digestibility coefficients
AI: adult intake
AOAC: Association of Official Analytical Chemists
ASVs: amplicon sequencing variants
BCFA: branched chain fatty acids
BHT: butil-hidroxitolueno
CP: crude protein
DM: dry matter
FEDIAF: European Pet Food Industry Federation
FDM: fecal dry matter
GE: gross energy
GTDB: Genome Taxonomy Database
LEfSe: linear discriminant analysis of effect size
ME: metabolizable energy
MER: metabolizable energy requirements
NRC: National Research Council
OM: organic matter
PCoA: principal coordinate analysis
PI: puppy intake
QIIME2: quantitative insights into microbial ecology
SCFA: short-chain fatty acids
TDF: total dietary fiber
ZR: Zymo Research
